# Establishing Patient Safety Cells in Public Tertiary Care Hospitals in India: A Practical Implementation Framework

**DOI:** 10.7759/cureus.110347

**Published:** 2026-06-06

**Authors:** Nitin Marathe, Rounak Dubey, Shilpa PN, Nikhila Bomma, Abin Varghese

**Affiliations:** 1 Hospital Administration, All India Institute of Medical Sciences, Nagpur, IND; 2 Transfusion Medicine, All India Institute of Medical Sciences, Nagpur, IND; 3 Nursing, All India Institute of Medical Sciences, Nagpur, IND

**Keywords:** clinical governance, patient safety, patient safety cell, public hospitals, quality improvement

## Abstract

Patient safety is recognised as a global health priority, and structured institutional mechanisms are required to reduce avoidable harm in healthcare settings. Public tertiary care hospitals in India manage high patient volumes, complex clinical services and resource constraints, all of which increase the need for coordinated patient safety governance. This technical report proposes a practical implementation framework for establishing Patient Safety Cells in public tertiary care hospitals in India, covering rationale, governance composition, core functions, a phased implementation pathway, patient safety indicators, incident reporting, root cause analysis (RCA) and failure mode and effects analysis (FMEA) workflows, integration with the Indian health system priorities, barriers, sustainability and minimum documentation. The novelty of the proposed model lies in consolidating fragmented safety activities under a single multidisciplinary operational hub explicitly aligned with the Indian public tertiary care environment and with various accreditation requirements. The expected institutional impact includes more consistent incident reporting and learning, structured root cause analysis and failure mode and effects analysis, accountable corrective action closure and stronger linkage between hospital-level practice and global and national patient safety priorities. Prospective implementation and evaluation are required to assess feasibility, effectiveness and long-term sustainability in routine public hospital practice.

## Introduction

Patient safety is now recognised as a fundamental component of universal health coverage and a global health priority [1]. The Seventy-second World Health Assembly adopted resolution WHA72.6 in 2019, which led to the development of the Global Patient Safety Action Plan 2021-2030, intended to provide strategic direction for eliminating avoidable harm in healthcare [1]. The Institute of Medicine report “To Err Is Human” first drew sustained international attention to the scale of preventable harm in hospitals more than two decades ago [2]. Analytical modelling of observational studies suggests that adverse events constitute a substantial share of the global burden of disease, and that low- and middle-income countries bear a disproportionately large share of harm [3]. The economic costs of unsafe care are also considerable, with estimates that direct treatment costs and downstream productivity losses run into trillions of dollars annually at a global level [4].

A system perspective on safety, exemplified by Reason’s analysis of human error, holds that errors typically emerge from latent conditions in complex systems rather than from individual failings [5]. This perspective has shaped contemporary patient safety practice, which emphasises non-punitive reporting, system redesign and organisational learning [5].

In India, the Ministry of Health and Family Welfare introduced the National Patient Safety Implementation Framework (NPSIF) 2018-2025 to provide a national platform for improving patient safety across all levels of care, and the Framework explicitly identifies the need for facility-level governance structures, adverse event reporting systems and a competent workforce to support patient safety [6]. The National Accreditation Board for Hospitals and Healthcare Providers (NABH) Accreditation Standards for Hospitals place patient safety at the centre of hospital quality assurance and require institutional structures for sentinel event review, incident reporting and corrective and preventive action [7]. Despite these enabling frameworks and accreditation standards, day-to-day institutional governance of patient safety in many public tertiary care hospitals remains fragmented across multiple statutory committees - typically infection control, pharmacovigilance, haemovigilance, quality assurance and grievance redressal - with limited dedicated capacity for integrated analytical work, learning and corrective action closure. This operational gap between the directions articulated in national patient safety frameworks and accreditation standards, and their institutional implementation, provides the rationale for the framework proposed in this technical report.

The Patient Safety Cell differs from existing quality and statutory committees in three important respects. First, in scope: while existing committees, such as infection control, pharmacovigilance, haemovigilance and quality assurance, each address a defined domain of risk, the Cell is the integrative operational unit that consolidates safety data from across these domains, coordinates institution-wide patient safety indicators and provides a single accountable forum for incident reporting and learning. Second, in function: the Cell owns and operationalises the analytical workflow - incident triage, root cause analysis, failure mode and effects analysis, corrective and preventive action tracking and structured feedback to reporting staff - rather than focusing primarily on accreditation documentation or domain-specific audit. Third, in governance: the Cell reports directly to the head of the institution, with explicit linkage to NABH and other applicable quality and accreditation standards, so that learning from incidents is translated into system-level corrective action rather than confined within the originating department [6,7].

A Patient Safety Cell is a dedicated multidisciplinary unit that consolidates institutional safety governance, incident reporting, root cause analysis, failure mode and effects analysis, training and indicator monitoring under one operational umbrella. This article proposes a practical and adaptable implementation framework for establishing such a cell in Indian public tertiary care hospitals, with explicit alignment to the WHO Global Patient Safety Action Plan, the NPSIF and NABH standards.

## Technical report

Definition and scope of a Patient Safety Cell

A Patient Safety Cell may be defined as a multidisciplinary institutional unit responsible for coordinating patient safety governance, incident reporting and analysis, corrective and preventive actions, safety training and monitoring of patient safety indicators across the hospital. The Cell does not replace existing statutory committees but serves as an operational hub that integrates safety-related work and reports to hospital leadership.

**Figure 1:**
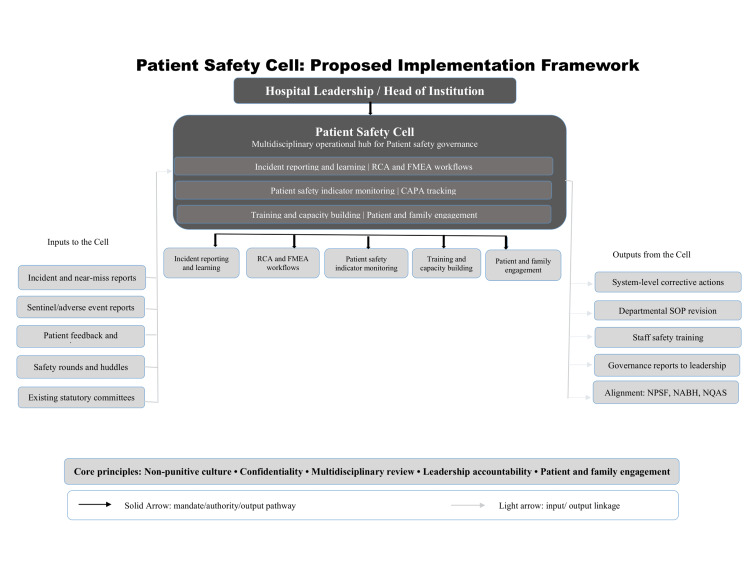
Proposed implementation framework for the Patient Safety Cell, illustrating the reporting relationship, core functions, inputs and outputs. SOPs: standard operating procedures; RCA: root cause analysis; CAPA: corrective and preventive action; Ayushman Bharat Digital Mission; NABH: National Accreditation Board for Hospitals and Healthcare Providers; NQAS: National Quality Assurance Standards; NPSIF: National Patient Safety Implementation Framework

The scope of a Patient Safety Cell is broad and includes incident reporting and learning systems, sentinel event review, root cause analysis and failure mode and effects analysis (FMEA), which is a prospective risk analysis technique adapted from engineering disciplines for healthcare use [8]. The Cell may facilitate medication safety, surgical safety, including implementation of the WHO Surgical Safety Checklist [9], and infection prevention and control activities aligned with WHO hand hygiene guidance [10]. It may also facilitate coordination with existing hospital-level and national safety reporting mechanisms, including pharmacovigilance, haemovigilance and materiovigilance. Additional domains may include diagnostic safety, patient handover, fall prevention, patient identification, safe injection practices, safe childbirth, patient and family engagement, staff training and monitoring of patient safety indicators. The overall framework, including the reporting relationship to the head of the institution, core functions, inputs from existing statutory committees and outputs, is illustrated in Figure 1.

Governance structure

The Patient Safety Cell should be constituted under formal institutional authority through an office order issued by the head of the institution. The proposed composition (Table 1) is illustrative and should be adapted to local staffing and organisational structure. A multidisciplinary composition is intended to capture inputs from all functional areas where patient harm can originate or where corrective actions must be implemented.

**Table 1:** Proposed Composition and Roles of a Patient Safety Cell HMIS: hospital management information system; RCA: root cause analysis; FMEA: failure mode and effects analysis; NABH: National Accreditation Board for Hospitals and Healthcare Providers

Member/position	Suggested designation	Key responsibilities
Chairperson	Medical Superintendent or Director	Provides institutional authority, chairs review meetings, signs off on corrective action plans and interfaces with the hospital governing body.
Nodal Officer/Member Secretary	Deputy Medical Superintendent or Faculty-in-Charge, Hospital Administration	Day-to-day operational responsibility, convenes meetings, coordinates RCA and FMEA, maintains documentation and submits reports.
Hospital Administrator/Quality Manager	Senior Hospital Administrator or NABH Coordinator	Aligns Cell activities with accreditation standards, supports indicator monitoring and coordinates audits.
Nursing Representative	Nursing Superintendent or Deputy	Represents nursing perspective, coordinates ward-level reporting and oversees nursing-related corrective actions.
Clinical Department Representatives	Faculty from Medicine, Surgery, Paediatrics, Obstetrics & Gynaecology, Anaesthesia, Emergency Medicine, Critical Care	Bring specialty-specific safety inputs, lead departmental RCAs and champion safety in their units.
Pharmacology/Pharmacy Representative	Faculty, Pharmacology and Chief Pharmacist	Coordinates medication safety, adverse drug reaction reporting and look-alike/sound-alike medication management.
Infection Prevention and Control Representative	Faculty, Microbiology/Hospital Infection Control Officer	Coordinates healthcare-associated infection surveillance, hand hygiene programmes and antimicrobial stewardship integration.
Blood Bank Representative	Officer-in-Charge, Blood Centre	Oversees haemovigilance, transfusion safety and transfusion reaction reporting.
Biomedical engineering representative	Biomedical Engineer	Coordinates equipment-related incident review, preventive maintenance gaps and device safety alerts.
IT/HMIS representative	Officer-in-Charge, IT/HMIS	Supports digital incident reporting workflows, dashboards and data linkage with the hospital information system.
Medical Records Representative	Officer-in-Charge, Medical Records	Supports documentation integrity, retrieval of case records for RCA, and audit-related data.
Resident Doctor Representative	Nominated Senior Resident	Represents the resident perspective, encourages frontline reporting and supports training.
Patient Relations/Grievance Representative	Officer-in-charge, Patient Relations/Grievance Cell	Brings patient-experience inputs and links grievance information with safety review where relevant.
Patient or Community Representative (where feasible)	Nominated patient or community member	Provides the patient and family perspective, supports patient and family engagement initiatives.

For operational consistency, the Cell may meet at a regular monthly frequency, with additional emergent meetings convened within 72 hours of a sentinel event or serious adverse event. A suggested quorum is at least 50% of nominated members, mandatorily including either the Chairperson or the Nodal Officer and at least one clinical department representative. Decisions, action items and target dates should be recorded in the minutes and reviewed at the next meeting through the corrective and preventive action (CAPA) tracker.

Patient and family engagement may be operationalised through several complementary mechanisms: periodic inclusion of patient or community representatives in Cell meetings as observers or invited participants; routine integration of patient grievance, feedback survey and complaint data into monthly safety reviews; structured discussion of anonymised patient stories or experiences at safety meetings to inform corrective actions; and use of patient-reported safety concerns as an additional input to incident review. Disclosure of unintended outcomes to patients and families, where applicable, should be coordinated through existing institutional disclosure and grievance pathways rather than created anew by the Cell.

Core functions of a Patient Safety Cell

The Patient Safety Cell should perform a defined set of core functions that together institutionalise patient safety governance. Patient safety governance includes the formal articulation of institutional safety policy, the approval of safety-related standard operating procedures, the periodic review of safety performance against indicators and accountability to hospital leadership. A robust incident reporting and learning system is the operational backbone of the Cell and requires simple, accessible reporting channels, time-bound review and structured feedback to reporting staff [11].

Near-miss reporting deserves particular attention because near misses are more frequent than harm events and offer learning opportunities without injury to patients [11]. Sentinel event review and root cause analysis allow the Cell to translate serious incidents into system-level countermeasures, with awareness that the strength of root cause analysis (RCA) recommendations is variable and benefits from structured oversight [12]. FMEA complements RCA by allowing the prospective identification and prioritisation of failure modes in high-risk processes before harm occurs [8].

The Cell should organise regular patient safety rounds in clinical and support areas, supported by short safety huddles in high-risk units. Dashboard-based monitoring of patient safety indicators enables longitudinal assessment and CAPA tracking ensures that recommendations are not lost between meetings. Training and capacity building, including induction of new staff and periodic refresher modules for clinical and non-clinical personnel, should be planned through an annual calendar. The Hospital Survey on Patient Safety Culture provides a structured instrument for assessing staff perceptions of patient safety culture and may complement technical safety interventions by tracking organisational culture [13]. Patient and family engagement, documentation and policy support and integration with infection control, pharmacovigilance, haemovigilance, biomedical equipment and other hospital committees complete the core function set.

Phased implementation framework

A phased approach allows the Patient Safety Cell to be established without disrupting existing services. The phases described in Table 2 are sequential but overlapping and can be adapted to local readiness. Timelines are indicative and should be calibrated to institutional capacity and to the existing accreditation status of the hospital.

**Table 2:** Phased Implementation Plan for Establishing Patient Safety Cells *In hospitals with limited HMIS/dashboard capacity, paper registers, standard formats, and Excel trackers may be used initially; digital dashboards can be developed progressively. SOP: standard operating procedure; RCA: root cause analysis; FMEA: failure mode and effects analysis; HMIS: hospital management information system; NABH; National Accreditation Board for Hospitals and Healthcare Providers; CAPA: corrective and preventive action

Phase	Key activities	Responsible persons	Expected outputs	Suggested timeline
1. Leadership approval and administrative mandate	Brief institutional leadership, obtain formal sanction, issue office order constituting the Cell.	Director/Medical Superintendent, Hospital Administration	Office order, terms of reference, and reporting line.	Month 1
2. Constitution of the Patient Safety Cell	Nominate multidisciplinary members, identify nodal officer, define meeting schedule.	Medical Superintendent, Nodal Officer	Constituted Cell with members and roles defined.	Months 1-2
3. Baseline safety assessment	Map existing committees, review previous incident records, and assess documentation gaps.	Nodal Officer; Quality Manager	Gap-analysis report, baseline indicator list.	Months 2-3
4. Development of the incident reporting system	Design simple paper- and HMIS-based reporting forms, define categories and severity grades, pilot in selected units.	Nodal Officer; IT/HMIS Representative	Incident reporting form, SOP, pilot data.	Months 3-5
5. Staff sensitisation and training	Conduct sensitisation sessions across cadres, emphasise a non-punitive culture and train champions.	Nodal Officer; Departmental Representatives	Training records, departmental champions identified.	Months 4-6
6. RCA and FMEA implementation	Train the core team in RCA and FMEA methods, initiate the first RCAs on sentinel events and pilot FMEA on a high-risk process.	Nodal Officer; Clinical Department Representatives	RCA and FMEA templates, first completed analyses.	Months 5-8
7. Patient safety dashboard and indicator monitoring	*Finalise indicator set, establish data flow, build dashboard, review monthly trends.	Nodal Officer; Quality Manager; IT/HMIS Representative	Patient safety dashboard, monthly indicator reports.	Months 6-9
8. Review meetings and feedback loop	Hold monthly Cell meetings, provide structured feedback to reporting staff and disseminate learning bulletins.	Chairperson, Nodal Officer	Meeting minutes, learning bulletins, and CAPA tracker updates.	Month 6 onwards
9. Integration with accreditation and hospital governance	Align Cell outputs with NABH and the requirements of other patient safety frameworks, link with statutory committees, report to the governing body.	Chairperson, Quality Manager	Integration map, periodic governance reports.	Months 9-12
10. Sustainability and annual safety reporting	Publish annual patient safety report; recognise good reporting practices; refresh training calendar; review terms of reference.	Chairperson; Nodal Officer	Annual patient safety report, recognition events, updated training plan.	Annually

Prioritisation across phases is important in resource-constrained settings. Phases 1 and 2 are strictly sequential prerequisites - leadership approval and formal constitution of the Cell must be in place before subsequent phases. Phases 3, 4 and 5 (baseline safety assessment, development of the incident reporting system and staff sensitisation and training) can be initiated in parallel once the Cell is constituted, allowing assessment, system design and capacity building to progress concurrently. Phase 6 (RCA and FMEA implementation) depends on adequate progress in Phases 4 and 5, because trained personnel and an operational reporting workflow are needed before structured analyses can begin. Phases 7-9 build progressively on these foundations and are expected to be substantially operational by the end of the first year. Phase 10 represents the annual sustainability cycle that sustains and reviews the Cell's functioning thereafter.

Patient safety indicators

A core set of patient safety indicators enables the Patient Safety Cell to support data-driven governance. Indicators may be selected according to institutional priorities, available data systems, service scope, risk profile and accreditation readiness, with reference to NABH or other applicable quality and patient safety frameworks. Suggested indicators may include medication errors, patient falls, healthcare-associated infections, surgical safety checklist compliance, adverse event reporting, sentinel events, needle-stick injuries, transfusion reactions, patient identification errors, hand hygiene compliance, pressure injuries and safe injection practices. Indicators should be operationally defined with clear numerators, denominators, responsible units and review frequencies. Hospitals seeking a ready reference for indicator definitions may draw on the Key Performance Indicators for patient safety monitoring specified in the NABH Accreditation Standards for Hospitals, 6th edition [7], which provide a structured and contextually applicable starting point for Indian public tertiary care settings.

Incident reporting and learning system

The incident reporting and learning system is the operational core of a Patient Safety Cell. A non-punitive reporting environment is the single most important precondition for adequate reporting volume and for honest description of events, and is consistent with the system-based view of error described by Reason [5]. Reporting forms should be short, structured and available both as paper formats and through the hospital information system to suit different work environments [11]. Anonymous reporting options can be offered for sensitive incidents where feasible, with the understanding that follow-up may be limited when anonymity is preserved.

Incidents should be categorised using clear operational definitions, distinguishing near misses, adverse events and sentinel events, and graded for severity. Time-bound review of reports, with an early acknowledgement to the reporting staff member and a defined window for analytical closure, helps to maintain trust in the system [11]. Monthly trend analysis allows the Cell to identify systemic issues that are not visible at the level of individual incidents. Periodic learning bulletins, anonymised and disseminated across departments, support institutional learning. A corrective action tracker, reviewed at each Cell meeting, ensures that recommendations are translated into action and closed within target timelines.

RCA and FMEA workflow

RCA and FMEA are complementary methods. RCA is retrospective and is applied to sentinel and serious events, while FMEA is prospective and is applied to high-risk processes before harm occurs [8]. Table 3 outlines a parallel step-wise workflow for RCA and FMEA under a Patient Safety Cell.

RCA should be triggered for all sentinel events and for any serious adverse event meeting institutional severity-grading criteria. Suggested triggers include unanticipated death not directly related to the natural course of the patient’s underlying illness; major permanent loss of function unrelated to the natural course; wrong-site, wrong-patient or wrong-procedure surgery; haemolytic transfusion reaction arising from ABO incompatibility; suicide of an admitted patient; infant abduction or discharge to a wrong family; retention of a foreign object after a surgical procedure; severe medication errors resulting in major harm; and maternal death directly related to the process of care. These categories are consistent with NABH-aligned definitions of sentinel events [7] and should be specified explicitly in the institutional patient safety policy so that triggering is consistent across cadres.

Caution is warranted in the application of RCA. Reviews have shown that many institutional RCAs yield weak recommendations and have limited measurable impact on safety outcomes when conducted in isolation or without adequate follow-up [12]. Methodological limitations have been highlighted, including the tendency to favour individual rather than system-level countermeasures and to address single incidents rather than recurrent patterns [14]. A systematic review of RCA in healthcare reported variable evidence for sustained safety improvement and emphasised the importance of strong action plans, follow-up and integration with the wider safety system [15]. These findings highlight the importance of conducting RCA as part of a coordinated Patient Safety Cell process, rather than treating it as a one-time or isolated activity.

**Table 3:** Suggested Workflow for RCA and FMEA Under a Patient Safety Cell SOPs: standard operating procedures; RCA: root cause analysis; FMEA: failure mode and effects analysis; CAPA: corrective and preventive action

Step	RCA workflow	FMEA workflow
Event selection	Identify a sentinel or serious adverse event meeting RCA criteria.	Select a high-risk or high-volume process for prospective analysis (e.g. medication administration, blood transfusion and patient transfer).
Team formation	Convene a multidisciplinary RCA team, including clinicians, nursing, pharmacy, and administration; nominate a facilitator.	Convene a multidisciplinary process team representing all stages and disciplines involved in the process.
Process mapping	Reconstruct a timeline of the event using records, interviews and observation.	Map the existing process step by step using flow diagrams.
Contributing factor/failure mode analysis	Identify contributing factors using structured frameworks (e.g. human factors, equipment, environment, organisational and communication).	For each step, identify potential failure modes, their causes, and downstream effects.
Risk prioritisation	Differentiate proximate causes from latent system factors, identify root causes.	Rate each failure mode for severity, occurrence, and detectability; prioritise using a hazard or risk priority score.
Action planning	Develop strong, system-level corrective actions; avoid over-reliance on training-only or policy-only solutions.	Design redesign actions, error-proofing, decision support, or process simplification for prioritised failure modes.
Implementation	Assign actions to identified owners with target dates, integrate with the CAPA tracker.	Pilot redesigned the process in a defined area before wider rollout.
Follow-up	Review the effectiveness of actions at defined intervals, re-open if recurrence is observed.	Monitor process indicators, reassess residual risk after implementation.
Documentation	Maintain RCA report, signed action plan, and meeting minutes in the Cell records.	Maintain FMEA worksheet, hazard ratings, and redesign documentation.
Learning dissemination	Anonymise and share lessons through learning bulletins and departmental briefings.	Share redesigned process and lessons with all relevant departments, update SOPs.

To illustrate operational application: An RCA triggered by an unanticipated cardiac arrest in a postoperative patient on the ward may involve a multidisciplinary team reviewing time-stamped vital signs documentation, escalation steps, response timing and equipment availability. Contributing factors might include delayed escalation due to ambiguous handover, absence of an early-warning-score chart at the bedside and limited staff training in recognising clinical deterioration. The resulting action plan might include implementation of a standardised early-warning-score chart, structured handover standard operating procedures (SOPs) and quarterly resuscitation drills, all tracked through the CAPA tracker.

A complementary FMEA example may be drawn from the blood transfusion process. The process team maps each step from sample collection to post-transfusion monitoring. Potential failure modes may include mislabelling of the patient sample at the bedside, a mismatch between the patient identification wristband and the issued blood unit at the time of administration, and inadequate post-transfusion observation. Each failure mode is rated for severity, occurrence and detectability, generating a risk priority score. High-priority failure modes may then be addressed through redesign actions such as barcode-based sample and unit verification, a structured two-person bedside identity check, a standardised post-transfusion observation chart and targeted retraining of transfusion staff. The same approach can be applied to high-alert medication administration, with failure modes such as look-alike/sound-alike confusion, incorrect dose calculation and inadequate double-check at administration.

Integration with the Indian health system priorities

The proposed framework aligns directly with the National Patient Safety Implementation Framework 2018-2025, which sets strategic direction for patient safety governance in India [6]. The NABH standards require institutional structures for quality and patient safety, sentinel event review, incident reporting and corrective and preventive action [7]. A Patient Safety Cell provides an effective mechanism through which these requirements can be operationalised. The National Quality Assurance Standards (NQAS) for public health facilities provide a structured quality assurance framework for public health facilities in India [16]. Vertical quality initiatives, such as LaQshya, focus on improving the quality of care in labour rooms and maternity operation theatres [17], while Kayakalp promotes cleanliness, hygiene, sanitation and infection-control practices in public health facilities [18]. These programmes provide complementary audit and improvement infrastructure that the Patient Safety Cell can leverage rather than duplicate.

Integration with the Ayushman Bharat Digital Mission (ABDM) and hospital information management systems may support digital incident reporting, indicator monitoring and longitudinal trend analysis where digital infrastructure is available [19]. The Cell also has an important academic role in tertiary care teaching hospitals, contributing to undergraduate, postgraduate and resident training in patient safety. Training activities coordinated by the Cell may be aligned with patient safety competencies articulated in national medical education frameworks for medical graduates and postgraduates, supporting the academic and capacity-building dimension of the Cell’s work alongside its operational functions. Public hospital resource limitations, including staffing constraints, high patient load and competing priorities, must be recognised in the design of any Cell, with care taken to add value rather than additional paperwork. State and central government institutions, including state health societies and apex public-sector teaching hospitals, can support diffusion of the model through training, mentorship and benchmarking.

Barriers and mitigation strategies

Several barriers may impede the establishment and functioning of Patient Safety Cells in public tertiary care hospitals. Table 4 summarises common barriers and practical mitigation strategies.

**Table 4:** Common Barriers and Practical Mitigation Strategies SOPs: standard operating procedures; RCA: root cause analysis; CAPA: corrective and preventive action; HMIS: hospital management information system; ABDM: Ayushman Bharat Digital Mission; NABH: National Accreditation Board for Hospitals and Healthcare Providers

Barrier	Likely impact	Practical mitigation strategy
Under-reporting of incidents	Loss of learning opportunities, inadequate trend data.	Simplify reporting forms, promote a non-punitive culture, provide visible feedback and recognise good reporting.
Fear of blame and litigation	Suppression of near-miss and adverse event reports.	Adopt and communicate an explicit non-punitive policy, protect confidentiality and focus reviews on system factors.
Limited staff awareness	Inconsistent reporting, weak departmental engagement.	Conduct structured sensitisation, induction modules, and departmental safety champions; integrate into resident teaching.
Staff shortage and competing service load	Inadequate time for reporting, RCA, and follow-up.	Embed reporting in routine workflows, use brief structured tools and prioritise a small number of high-impact indicators.
Inadequate documentation	Difficulty in case reconstruction during RCA.	Strengthen medical record documentation standards, integrate with NABH-aligned audits.
Fragmented data systems	Inability to link incidents with denominators or with other safety data.	Leverage HMIS and ABDM-enabled tools, build a single patient safety dashboard, standardise indicator definitions.
Lack of feedback after reporting	Erosion of trust in the reporting system.	Acknowledge each report within a defined window, share anonymised lessons, report periodic indicator updates.
Weak corrective action closure	Recurrence of similar incidents.	Maintain a CAPA tracker reviewed at every Cell meeting, assign clear owners and target dates.
Limited leadership engagement	Loss of institutional priority, difficulty in implementing system-level changes.	Embed the Cell within the senior management structure, provide periodic reports to the governing body.
Competing service priorities	Patient safety work deprioritised during clinical surges.	Define minimum sustainable activities (monthly meeting, indicator review) that continue even during high load.
Poor interdepartmental coordination	Recommendations remain confined within the originating department.	Use the Cell as a cross-functional forum, circulate minutes, standardise SOPs across departments.

Sustainability model

Sustainability of a Patient Safety Cell depends on enduring institutional commitment rather than time-limited project activity. Leadership commitment, expressed through visible participation of the head of the institution in periodic reviews, is a foundational element. An administrative mandate, reflected in an updated terms-of-reference document and an annual office order, anchors the Cell within the organisational structure. Monthly review meetings with structured minutes and tracked actions create a predictable rhythm and accountability.

Departmental patient safety champions distribute the work and signal that patient safety is a shared responsibility, while a sustained non-punitive culture is essential for honest reporting [5]. A formal annual training calendar, covering incident reporting, RCA, FMEA, hand hygiene [10], surgical safety [9] and medication safety, supports the continuous development of staff. Dashboard-based monitoring with periodic public reporting within the hospital reinforces transparency. An annual patient safety report, published internally and shared with the governing body, summarises performance and informs priorities for the next cycle. Integration with NABH and NQAS requirements ensures that the Cell’s work also serves accreditation and external assessment processes [7,16]. Recognition of good reporting practices, use of hospital management information system (HMIS)- and ABDM-enabled digital tools and continuous improvement cycles round out a sustainable model.

Proposed minimum documentation set

A minimum documentation set defines the records that should be maintained by the Patient Safety Cell to support governance, audit and learning. Table 5 lists suggested documents, their purpose, frequency of update and the responsible person.

**Table 5:** Minimum Documentation Set for a Patient Safety Cell RCA: root cause analysis; FMEA: failure mode and effects analysis

Document/record	Purpose	Frequency of update	Responsible person
Patient safety policy	Articulates institutional commitment, scope, and principles, including non-punitive reporting.	At least every three years or on a regulatory update	Chairperson
Incident reporting form	Captures structured information on adverse events and unsafe conditions.	Continuous use; reviewed annually	Nodal Officer
Near-miss reporting form	Captures near misses and good catches.	Continuous use; reviewed annually	Nodal Officer
Sentinel event register	Maintains a chronological record of sentinel events with the status of review.	Updated on each event	Nodal Officer
RCA template	Structures the analytical workflow for sentinel and serious events.	Updated as needed	Nodal Officer
FMEA template	Structures prospective risk analysis of selected processes.	Updated as needed	Nodal Officer
Corrective and preventive action (CAPA) tracker	Tracks recommendations to closure with the owner and target date.	Reviewed at every Cell meeting	Nodal Officer
Patient safety dashboard	Visual summary of safety indicators with trends.	Monthly	Quality Manager/IT representative
Training register	Records all patient safety training activities and attendees.	Updated after each training	Nodal Officer
Meeting minutes	Documents discussions, decisions, and action items.	Each meeting	Member Secretary
Safety round checklist	Structures patient safety rounds in clinical and support areas.	Updated annually; used each round	Nodal Officer
Annual patient safety report	Summarises annual performance, key learnings and priorities for the next cycle.	Annual	Chairperson and Nodal Officer

## Discussion

The proposed Patient Safety Cell framework provides a structured approach to institutionalising patient safety in public tertiary care hospitals in India. It aligns with the strategic objectives of the WHO Global Patient Safety Action Plan 2021-2030, particularly those addressing high-reliability systems, safety of clinical processes, health worker education and information and learning [1]. Experience from other settings, including the United States Department of Veterans Affairs National Center for Patient Safety, demonstrates that structured patient safety tools, such as Healthcare Failure Mode and Effect Analysis, can support prospective identification and mitigation of risks in healthcare processes [8].

Comparison with global priorities indicates that several elements emphasised in the framework - non-punitive reporting, structured RCA and FMEA, surgical safety, medication safety and hand hygiene - correspond to established international evidence and guidance [9,10]. Tracking patient safety culture using validated instruments, such as the Hospital Survey on Patient Safety Culture, can help the Cell identify whether technical interventions are translating into sustained behavioural and organisational change, providing a longitudinal perspective that complements the operational safety data generated through incident reporting and indicator review [13]. Vincent and Amalberti have argued that safety strategies must be matched to the operational realities of different care environments, including those with high variability and resource constraints [20]. Public tertiary care hospitals in India represent precisely such complex environments, and the Patient Safety Cell framework is intended to be feasible within these constraints rather than to depend on idealised conditions.

In resource-constrained settings, this framework can strengthen clinical governance by consolidating fragmented quality and safety activities, generating actionable data and establishing a single accountable institutional unit. Where applicable, it may also support compliance with the NABH standards and other relevant quality and patient-safety framework requirements [6,7].

In this context, the governance composition and phased implementation pathway described in this framework are structured to work within existing institutional roles and available infrastructure, without requiring dedicated full-time appointments or advanced digital systems as prerequisites for operationalisation. Future comparative research examining institutional patient safety governance models across Indian and other low- and middle-income country settings would further inform contextual adaptation of this framework.

However, the framework may support - but does not by itself guarantee improvements in patient outcomes. Its effectiveness will depend on leadership commitment, a sustained non-punitive culture, appropriate staffing of the Cell and integration with the wider quality ecosystem. Therefore, the framework is proposed as a structured and adaptable approach rather than a prescriptive blueprint. Contextual modification is essential, and prospective evaluation is required to establish its operational and clinical impact.

Limitations

This article presents a practical implementation framework and does not include primary data or evaluation of implementation outcomes. The proposed framework is intended to be adaptable and may require modification according to local staffing, infrastructure, digital systems, governance arrangements and accreditation status. As the framework has not yet been prospectively tested, its feasibility, effect on safety culture, influence on incident reporting trends and impact on patient outcome indicators should be evaluated in diverse public tertiary care settings. Empirical quantification of patient safety incidents, adverse events and existing governance practices in Indian public tertiary care hospitals - ideally through multi-centre studies and a dedicated synthesis of published Indian evidence - is an important direction for future research, and would complement the implementation framework proposed here.

## Conclusions

Patient Safety Cells can provide a structured institutional mechanism for strengthening patient safety governance in public tertiary care hospitals in India. By supporting incident reporting and learning, root cause analysis, failure mode and effects analysis, patient safety indicator monitoring and follow-up of corrective and preventive actions, these Cells can help convert patient safety from an individual responsibility into an organised institutional function. The proposed framework may help public tertiary care hospitals organise patient safety activities into a more structured, multidisciplinary and accountable institutional process. Future empirical evaluation across diverse public tertiary care settings is required to assess the effectiveness, sustainability and impact of Patient Safety Cells in routine hospital practice.
